# Association of analgesic pharmacological effect with pain site in pediatric oncology patients

**DOI:** 10.3389/fpain.2026.1685357

**Published:** 2026-03-04

**Authors:** Flavia Carvalho Marotta, Eduardo Ladeia Leal, Isabelle Alvarenga Oliveira, Thuane Sales Gonçalves, Silmara Rodrigues Machado, Carolina Paula Jesus Kasa, Paulo Caleb Júnior Lima Santos

**Affiliations:** 1 Department of Pharmacology, Escola Paulista de Medicina, Universidade Federal de São Paulo, São Paulo, Brazil; 2 Grupo de Apoio ao Adolescente e Criança com Câncer, Universidade Federal de São Paulo, São Paulo, Brazil; 3 Sociedade Beneficente de Senhoras, Hospital Sírio-Libanês, São Paulo, Brazil

**Keywords:** pediatric oncology, pain, dipyrone, morphine, pharmacologic effects

## Abstract

**Background:**

Cancer in children and adolescents is frequently associated with pain, which is one of the most common and distressing symptoms reported by patients. Effective pain management remains a major concern for healthcare teams. Despite the availability of national and international pain management protocols since the mid-1980s, challenges persist in the assessment, treatment, and follow-up of pediatric patients. There is a lack of studies evaluating the most appropriate type and dosage of analgesics to achieve adequate pain control in pediatric oncology settings.

**Objective:**

The objective of this work was to assess the effectiveness of selected analgesics based on pain intensity and anatomical location in pediatric cancer patients.

**Methods:**

This pharmacoepidemiological study was conducted in a pediatric oncology hospital and included patients aged between 0 and 17 years with cancer who received analgesic drugs. Information regarding cancer diagnosis, hospitalization diagnosis, analgesic scale, pain intensity before and after drug administration, and pain site were collected from medical records.

**Results:**

A total of 1,465 episodes of pain from 335 patients were analyzed, most of them in patients diagnosed with leukemia (30.1%). We included 576 episodes of pain treated with dipyrone or morphine, occurring in the abdomen (*n* = 283), head (*n* = 155), and lower limbs (*n* = 138). The final pain scores indicated pharmacological effectiveness in all patient subgroups. When pain was mild to moderate, dipyrone was the most commonly used drug: 105 (65.2%) episodes of pain that occurred in the abdomen, 93 (86.9%) in the head, and 50 (64.1%) in the lower limbs. However, when the pain was severe to unbearable, morphine was the most commonly used drug: 79 (64.7%) episodes in the abdomen and 36 (60.6%) in the lower limbs, except when the pain occurred in the head (17 episodes of pain, 35.4%).

**Conclusions:**

The use of dipyrone and morphine, guided by pain intensities and locations, demonstrated effectiveness. These findings support the tailored use of analgesics according to pain characteristics to optimize symptom control in pediatric oncology patients.

## 1 Introduction

Each year, an estimated 400,000 children and adolescents worldwide are diagnosed with cancer (1). Currently, in developed countries, approximately 80% of youth cancer cases can be cured if diagnosed and treated early. Despite advances in youth cancer treatment, health teams remain deeply concerned with the care of these patients. One area of care is pain management, which requires assessment, intervention (pharmacological or otherwise), and subsequent reassessment (2–4).

In 2020, the International Association for the Study of Pain (IASP) updated its definition of pain as “an unpleasant sensory and emotional experience associated with, or resembling that associated with, actual or potential tissue injury.” The evaluation and rapid treatment of pain therefore depend on a trained and prepared multidisciplinary team (5).

A systematic review indicated a pain prevalence of 44.5% among cancer patients (6). Among those undergoing treatment, 55% report pain, while 66% of patients with advanced, metastatic, or end-stage cancer report this symptom. Multiple physiological factors contribute to cancer-related pain, such as the presence of the tumor in tissue, whether the tumor compresses the tissue, and the adverse effects of treatment, including peripheral neuropathy, muscle spasm, constipation. In addition, feelings of pain exert a negative psychological and social impact, extending to family members (7–9).

There are several validated tools to measure pain, classifying intensity both qualitatively and quantitatively, the latter via scores. When self-reporting is possible, a numerical scale between 0 and 10 can be used, with 0 representing the absence of pain and 10 representing unbearable pain. Alternatively, a diagram with facial representations of different levels of pain can be used. In cases where self-reporting is not possible, an assessment of behavior and physiological aspects indicative of pain is used, such as the Face, Legs, Activity, Cry, and Consolability (FLACC) scale (10–14).

The WHO has proposed an analgesic ladder for pain management, consisting of three steps. The first step addresses mild pain with pharmacological treatment of non-opioid analgesics, such as non-steroidal anti-inflammatory drugs or paracetamol, which may include an adjuvant drug. The second step targets moderate pain and includes weak opioids (codeine and tramadol), along with the treatment proposed in the first step. The third and final step addresses intense and unbearable pain, adding a strong opioid (such as morphine, fentanyl, methadone, or oxycodone) to the drugs in the second step. In 2023, this analgesic ladder was updated to include a fourth step, incorporating non-pharmacological procedures and techniques (15). The analgesic ladder is only a general guide to pain management; consequently, individual countries or regions, such as Europe (ESMO 2010), develop their own guidelines tailored to their respective realities. For effective control, it is essential to adopt individualized approaches that consider the child's stage of development and social context, valuing safety when using medication, through multiprofessional actions and pharmaceutical care (4, 16–18). Pain is a highly prevalent symptom in pediatric cancer patients, but its management is still often inadequate (19). Despite its prevalence, there is a paucity of comprehensive studies evaluating dosage and appropriate selection of analgesics to achieve an optimal therapeutic effect in these patients (20). Given this gap, there is a significant opportunity to improve pain management strategies in pediatric oncology. Thus, the main aim of the present study was to evaluate the effectiveness of the chosen analgesic medication according to the intensity and location of pain.

## 2 Materials and methods

This retrospective descriptive pharmacoepidemiological study was conducted at Hospital GRAACC (Grupo de Apoio ao Adolescente e Criança com Câncer—Ethics Committees: 64195522.8.0000.5505 and IOP-007-2020), a children's oncology hospital located in São Paulo, Brazil. Pediatric patients aged between 0 and 17 years, diagnosed with cancer and admitted to the hospital between January 2021 and March 2022, were eligible for inclusion in this study.

During this period, 905 prescriptions involving analgesic drug were selected. From the sample of patients, individuals with meticulously documented pain records, containing the intensity of pain before and after reassessment, the scale used in both assessments, the name of the administered medication as well as the dose and the route of administration, and the time between the two assessments were considered for inclusion (*n* = 355 patients with 1,465 episodes of pain).

Each patient was assessed for pain by a nurse, who used one of these three methods: FLACC scale, facial scale, or numerical scale. All nurses received training on choosing a pain assessment instrument and its correct use. The intensity of pain scale was standardized from 0 to 10, with 0 representing no pain and 10 representing the most intense pain that can be felt. Self-reporting of pain was prioritized, and when this was not possible, a validated instrument independent of the patient's self-report was applied to measure pain. After obtaining the numerical intensity of the patient's pain, it was recorded in the patient's specific medical record, along with the indication for pharmacological or non-pharmacological intervention for pain relief.

After the intervention for pain relief, the patient was reassessed within 30 min, in accordance with the institutional guidelines, using the same instrument to identify pain relief or determine the need for a new intervention.

The choice and subsequent dispensation of analgesic medications followed the institutional pain management protocol. According to this protocol, the drugs dipyrone and paracetamol are recommended for mild pain; tramadol in cases of moderate to severe pain; and morphine for severe or unbearable pain. The dosing regimen depended on the patient's body weight, and the frequency of administration was in accordance with established guidelines on serum drug concentrations.

We included those who underwent pain assessment using a scale and obtained a score ≥1, who then received paracetamol, dipyrone, ketorolac, morphine, nalbuphine, or tramadol for pain management, and who underwent pain reassessment after pharmacological intervention.

From medical records, data were collected on patient age, gender, cancer diagnosis, the tool used to assess pain, the intensity score before and after pharmacological intervention, and the time interval between reassessments. Regarding the drugs used, information on the name of the drug, dosage, and route of administration was collected.

Data on cancer diagnosis were grouped according to the International Classification of Childhood Cancer (ICCC). The ICCC classifies childhood neoplasms into 12 main groups, which are subdivided into 47 subgroups. The 12 main groups are as follows: (I) leukemias, myeloproliferative and myelodysplastic diseases; (II) lymphomas and reticuloendothelial neoplasms; (III) central nervous system (CNS) tumors and miscellaneous intracranial and intraspinal neoplasms; (IV) tumors of the sympathetic nervous system; (V) retinoblastoma; (VI) renal tumors; (VII) liver tumors; (VIII) malignant bone tumors; (IX) soft tissue sarcomas; (X) germ cell, trophoblastic, and other gonadal neoplasms; (XI) carcinomas and other malignant epithelial neoplasms; and (XII) other unspecified malignant tumors (21). The age of the patients was categorized according to development, considering infants as those aged between 0 and 1 year; preschoolers, aged between 2 and 4 years; schoolchildren, aged between 5 and 10 years; and adolescents, aged between 11 and 19 years. The pain intensity score was grouped as “no pain” when it was equal to 0, “mild” from 1 to 3, “moderate” from 4 to 6, “severe” from 7 to 9, and 10 for “unbearable.” The reassessment score was grouped in relation to the first score: A final score of 0 meant a total improvement for the patient, with no pain complaints; a final score lower than the initial score meant a partial improvement; a final score equal to the initial score meant no improvement; and a final score higher than the initial score meant a worsening of pain. Each report of pain or pain condition verified by the nursing or medical team was considered a pain episode.

### 2.1 Statistical analysis

The data were described as mean and standard deviation or median for continuous variables and as count and percentage for categorical variables. Normality was tested using the Kolmogorov–Smirnov test. Comparisons between initial and final pain scores were determined using paired measures Wilcoxon test. Chi-square test was used to compare the differences in the proportion of drugs used according to the location and intensity of the pain. As this was a retrospective observational study, no *a priori* sample size calculation was performed. All statistical analyses were conducted with the SPSS version 27.0 statistical package (22).

## 3 Results

A total of 1,465 episodes of pain from 335 patients were included, most of whom were male patients (55.5%). The predominant age groups were school-aged children and adolescents, which included patients between 5 and 19 years, totaling 227 individuals and representing 67.7% of the patients. According to the International Classification of Childhood Cancer (ICCC), the most frequent diagnoses were “leukemias, myeloproliferative and myelodysplastic diseases” (*n* = 101; 30.1%), followed by “central nervous system (CNS) tumors and miscellaneous intracranial and intraspinal neoplasms” (*n* = 76; 22.7%) (Supplementary Table S1 presents the general characteristics of patients).

Each report of pain by the patient, or verified by the team, was considered an episode of pain, and these reports were grouped by pain sites, as described in Table 1.

**Table 1:** Number of pain episodes by site.

Site	Abdomen	Head	MMII	Widespread	MMSS	Other	Thorax	Total
Pain episodes	312	184	154	43	24	731	17	1,465

MMII, lower limbs; MMSS, upper limbs. Other was composed of anal, lumbar, painful urination, bladder, mouth, catheter in external jugular vein, cervical, menstrual cramps, back, tooth, dorsal, epigastric pain, scapula, sternum, stomach, left face, right flank, throat, throat and ear, large bones, surgical incision, catheter insertion, tongue, lumbar and cervical, jaw, upper limbs and lower limbs, unspecified, shoulder, shoulders, shoulders and mouth, ear, pelvis, perianal, perineum, hip, pelvic region, penile region, suprapubic, and testicles. Pain sites categorized as “others” include 42 locations. Each of these locations represented fewer than 100 pain episodes and was subsequently excluded from the study due to low statistical power.

Sites of generalized pain, upper limbs, and thorax were excluded due to a low sample size. For episodes of pain in the abdomen, head, and lower limbs (*n* = 650), the drugs dipyrone, paracetamol, ketorolac, morphine, tramadol, and nalbuphine were given as pharmacological treatment. Due to the low sample size, those episodes of pain that received paracetamol, ketorolac, tramadol, and nalbuphine as treatment were excluded (*n* = 74). Thus, 576 episodes of pain (39.3%; 576/1,465) were considered. These were treated with dipyrone or morphine: 283 (19.3%) in the abdomen, 155 (10.6%) in the head, and 138 (9.4%) in the lower limbs. The most frequent route of administration was intravenous (IV) for both dipyrone (98.6%) and morphine (97.8%) treatment.

**Figure 1:**
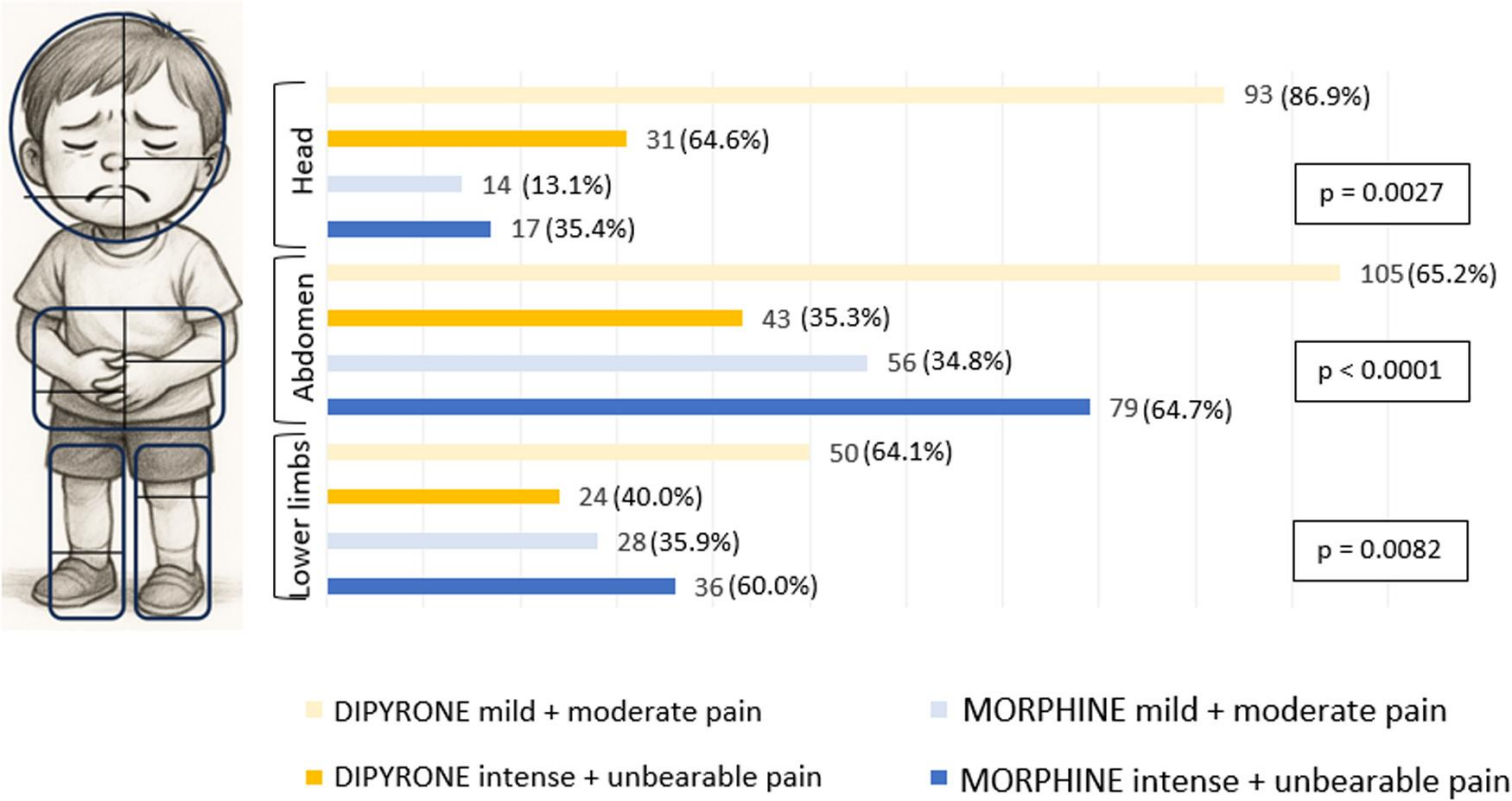
Frequencies of drugs used according to pain location and intensity.

To evaluate the analgesic effect of morphine and dipyrone, we verified how many episodes of pain were treated with these drugs for each site, stratifying the intensity into two groups: one group composed of mild to moderate (*n* = 346; 60%) and the other of intense to unbearable (*n* = 230; 40%) (Figure 1).

In pain considered mild to moderate, dipyrone was preferred to morphine. When the pain was stronger (intense to unbearable), there was a preference for the morphine when the pain was in the abdomen and lower limbs. In terms of intense to unbearable pain, dipyrone was more commonly used than morphine in cases of pain in the head region (Figure 1). In this study, each pain episode was evaluated individually. Pain intensity was scored both before and after the administration of the analgesic (dipyrone or morphine). The interval between these two assessments ranged from 10 to 30 min in 82% of the pain episodes, which is consistent with the anticipated average time for the onset of analgesia.

To verify the effectiveness of the chosen medication, we compared pain intensity scores before and after pharmacological intervention. In all situations, the median initial score was significantly different and higher than the median final score, confirming that the drugs dipyrone and morphine decreased pain in a noticeable way, improving the patient's state in relation to pain (Tables 2, 3).

**Table 2:** Pharmacological effectiveness according to drug, location of pain, and intensity.

Site	Abdomen		Head		Lower limbs	
Median score	Median initial score	Median final score	Median initial score	Median final score	Median initial score	Median final score
Dipyrone*						
Mild + moderate	5	0	4	0	5	0
Severe + unbearable	8	0	8	0	8	0
Morphine*						
Mild + moderate	5	0	5	0	5	0
Severe + unbearable	8	0	8	0	8	0

* *P* < 0.001 for all comparisons between initial and final median scores.

**Table 3:** Relative frequency (%) of pain episodes with total improvement.

Location of pain	Abdomen (%)	Head (%)	Lower limbs (%)
Dipyrone			
Mild + moderate	96.2	97.8	96.0
Severe + unbearable	83.7	77.4	75.0
Morphine			
Mild + moderate	91.1	92.9	92.9
Severe + unbearable	87.3	76.5	97.2

## 4 Discussion

The choice of pharmacological treatment was effective when considering both pain intensity and location. Notably, when the pain was located in the head, dipyrone was more frequently administered across both pain intensity groups. The most prevalent cancer types among children and adolescents in this study were leukemias, myeloproliferative disorders, and myelodysplastic syndromes, accounting for 30.1% of cases. In Brazil, the most prevalent cancer in this population is leukemia (26%), followed by lymphomas (14%) and central nervous system tumors (13%) (23).

This study revealed a higher incidence of mild and moderate pain, consistent with the findings of Andersson et al. (24), who reported a greater occurrence of mild and moderate pain both at rest and during movement. However, this contrasts with the findings of Bakir et al. (25), whose study noted that severe pain was present in 72.2% of cancer patients under 18 years of age admitted to a clinic. This difference may be due to the fact that their study was conducted in a clinic that specialized in chronic pain.

The most frequently reported pain location in this study was the abdomen, followed by the head and lower limbs. Similarly, Bakir et al. also found a higher incidence of pain in these three regions. However, in their study, the most common location was the lower limbs, followed by the abdomen and head.

In the present study, dipyrone was administered in 60% of pain episodes and morphine in 40%. Another study, conducted in the pediatric division of a teaching hospital in São Paulo, observed that dipyrone was administered in 76.1% of children experiencing pain. Among children with severe pain (125 cases), only 18 received morphine. That study did not assess the effectiveness of the analgesics, i.e., whether pain was reduced after administration, likely due to a low percentage of recorded pain reassessments (40.3%) (26).

In a study by Anderson et al., 74% of patients reported, via a questionnaire, that the administered analgesics—either alone or in combination—relieved their pain. These included paracetamol, nonsteroidal anti-inflammatory drugs (NSAIDs), and opioids. These findings are consistent with the present study, in which pain relief was also significant (24). Dipyrone, although widely used in Brazil as a first-line analgesic, is banned in the United States and in several other countries that contribute substantially to the pediatric pain literature. This regulatory difference limits the availability of international studies involving dipyrone and may contribute to methodological approaches that differ from those commonly reported.

It is important to note that 78% (*n* = 455) of the pain episodes involved associated analgesic treatment, which may have included one or more agents from the following classes: NSAIDs, opioids, corticosteroids, anticonvulsants, antidepressants, and/or anxiolytics. This practice demonstrates that the associated treatment may be related to the type of pain (neuropathic or nociceptive) and to strategies aimed at reducing the adverse effects of the medications used. Despite patients already receiving other analgesics or class of medication, additional pharmacological interventions were often required at the initial assessment of the pain episode.

Pain management in children with cancer presents multiple challenges, including difficulty in evaluating analgesic effectiveness, the persistent of multifactorial pain, and frequent treatment with multiple interventions. Therefore, the present study has limitations when it comes to observing all aspects of the pain scenario. First, the scarcity of randomized clinical trials with high levels of evidence undermines the scientific foundation of therapeutic approaches. Pain control is often based on less robust evidence, such as case reports and case series, and it is common for pediatric analgesic dosages to be extrapolated from adult-based guidelines (27). Second, when a pain complaint treated with medication was reassessed without improvement, additional interventions were carried out to ensure better analgesia for the patient. Furthermore, since the study evaluated each pain episode in isolation, it was not possible to determine which strategy was applied in each case—whether a dose adjustment or a change in the analgesic agent. Third, we did not include certain variables such as improvements in sleep quality, physical activity, or diet. In addition, information regarding the occurrence of adverse effects related to the medications used was not assessed. Fourth, this study grouped pain intensity differently from other reports. Most studies classify the pain scale as follows: 0 = no pain, 1–3 = mild pain, 4–6 = moderate pain, and 7–10 = severe pain. However, the present study considered a score of 10 to represent unbearable pain.

Pain can be classified into three main categories: nociceptive, neuropathic, and mixed. Distinguishing between these etiologies requires careful clinical evaluation, often supported by standardized tools such as validated questionnaires designed to identify the type of pain. However, given that the study population included children aged between 0 and 17 years, and that no validated instruments exist for the assessment of neuropathic pain in children under 5 years of age (28), it was not possible to accurately differentiate pain types among the participants.

Finally, this study is a real-world evaluation that did not stratify patients according to the stage of diagnosis, time of reassessment after drug administration, and method of drug administration.

## 5 Conclusions

The use of dipyrone and morphine, guided by pain intensities and locations, proved effective in reducing pain. These findings support the tailored use of analgesics according to pain characteristics, thereby optimizing symptom control in pediatric oncology patients.

## Data Availability

The raw data supporting the conclusions of this article will be made available by the authors without undue reservation.
