# Supplementary material for: Association of analgesic pharmacological effect with pain site in pediatric oncology patients
Source: Front Pain Res (Lausanne). 2026 Mar 4;7:1685357. doi: 10.3389/fpain.2026.1685357 (PMC12996144; doi:10.3389/fpain.2026.1685357)
Supplement: Supplementary file 1 [file Table1.pdf]

Supplementary Table 1. General characteristics total group and sub-group of patients.

Group	Total group		Sub-group*	
**Absolute and relative numbers**	N (n = 335)	%	n (n = 184)	%
**Sex**				
Female	149	44.5	84	45.5
Male	186	55.5	100	54.5
**Age Range**				
Infant	37	11.1	11	5.9
Preschool	71	21.2	36	19.6
School	112	33.4	66	35.9
Adolescent	115	34.3	71	38.6
**International Classification of Childhood Cancer**				
Leukemias, Myeloproliferative, and Myelodysplastic Diseases	101	30.1	67	36.4
Lymphomas and Reticuloendothelial Neoplasms	21	6.3	12	6.5
CNS and Miscellaneous Intracranial and Intraspinal Neoplasms	76	22.7	38	20.7
Neuroblastoma and Other Peripheral Nervous Cell Tumors	24	7.2	14	7.6
Retinoblastoma	21	6.2	12	6.5
Renal tumors	25	7.5	8	4.4
Hepatic tumors	1	0.3	0	0
Malignant Bone Tumors	35	10.4	16	8.7
Soft Tissue and Other Extraosseous Sarcomas	20	6.0	10	5.4
Germ Cell Tumors, Trophoblastic Tumors, and Neoplasms of Gonads	1	0.3	0	0
Other Malignant Epithelial Neoplasms and Malignant Melanomas	4	1.2	4	2.2
Other and Unspecified Malignant Neoplasms	6	1.8	3	1.6

*Sub-group mean patients with pain episodes in head, abdomen and lower limbs, witch ones were treated with dipyrone or morphine
